# Extracellular Vesicles in Viral Pathogenesis: A Case of Dr. Jekyll and Mr. Hyde

**DOI:** 10.3390/life11010045

**Published:** 2021-01-13

**Authors:** Lada Purvinsh, Andrey Gorshkov, Aleksandra Brodskaia, Andrey Vasin

**Affiliations:** 1 Institute of Biomedical Systems and Botechnologies, Peter the Great St. Petersburg Polytechnic University, 194021 St. Petersburg, Russia; ladapurvinsh13@gmail.com (L.P.); a.brodskaya1988@gmail.com (A.B.); vasin_av@spbstu.ru (A.V.); 2 Department of Molecular Biology of Viruses, Smorodintsev Research Institute of Influenza, Ministry of Health of the Russian Federation, 197376 St. Petersburg, Russia

**Keywords:** extracellular vesicles, viral pathogenesis, antiviral immune response, microRNA

## Abstract

Secretion of extracellular vesicles (EVs) is a fundamental property of living cells. EVs are known to transfer biological signals between cells and thus regulate the functional state of recipient cells. Such vesicles mediate the intercellular transport of many biologically active molecules (proteins, nucleic acids, specific lipids) and participate in regulation of key physiological processes. In addition, EVs are involved in the pathogenesis of multiple diseases: infectious, neurodegenerative, and oncological. The current EV classification into microvesicles, apoptotic bodies, and exosomes is based on their size, pathways of cellular biogenesis, and molecular composition. This review is focused on analysis of the role of EVs (mainly exosomes) in the pathogenesis of viral infection. We briefly characterize the biogenesis and molecular composition of various EV types. Then, we consider EV-mediated pro- and anti-viral mechanisms. EV secretion by infected cells can be an important factor of virus spread in target cell populations, or a protective factor limiting viral invasion. The data discussed in this review, on the effect of EV secretion by infected cells on processes in neighboring cells and on immune cells, are of high significance in the search for new therapeutic approaches and for design of new generations of vaccines.

## 1. Introduction

Intense intercellular communication, providing functional integration of cells and tissues in the organism, is the main attribute of multicellular life forms. In recent decades, the rapid progress of high-performance –omics technologies in biology has led to a secretome concept, as a sum of biologically active substances, of various natures, secreted by the cell, including soluble molecules, various granules, and extracellular vesicles. Secretome components circulate in the local intercellular medium or they can be transported by biological fluids to recipient cells throughout the organism. The cellular secretome determines the functional tuning of other cells in the organism [1] and also plays a key role in the pathogenesis of many diseases, among them infectious diseases. Besides molecules, significant portions of the cell secretome are more complex components, including various nanoparticles and several types of extracellular vesicles [2]. The phenomenon of membrane vesicle secretion by cells was first discovered more than three decades ago [3]. Extracellular vesicles were found to be secreted by all types of cells, and they were detected in various biological fluids: urine [4], blood [5], saliva [6], breast milk [7], bronchoalveolar fluid [8], amniotic fluid [9], and others.

Different types of extracellular vesicles (EVs) have certain degrees of similarity in physical and morphological characteristics (size, shape, buoyant density). Therefore, for classification of EVs, a complex of attributes is used, including pathways of biogenesis, size, and molecular composition [10]. The molecular composition of EVs depends on the type and functional state of the cells secreting them, and there are mechanisms that allow certain cellular proteins, lipids, and nucleic acids to be selectively transported into various types of vesicles. Currently, there are three databases accumulating information on the molecular composition of extracellular vesicles: EVpedia [11]; Vesiculepedia [12]; and Exocarta [13].

Over the past two decades, the role of EVs has been shown in several important biological processes such as immunosuppression during trophoblast implantation and placenta formation [14]; antigen presentation [15]; and proinflammatory cytokine secretion [16]. EVs play a key role in the pathogenesis of many diseases, among them oncological diseases [17,18,19], amyloidosis, and prion infection [20,21]. Therefore, assessment of changes in molecular EV composition (particularly, exosomal microRNA composition) can be a biomarker for various pathological states of cells and potentially an important diagnostic and prognostic tool in medicine.

Extracellular vesicles (especially exosomes) have been confirmed to be actively involved in the pathogenesis of various viral diseases [22,23,24]. Studies of the composition of EVs secreted by virus-infected cells has revealed that EVs can specifically pack and transport not only cellular proteins and nucleic acids, but also viral components and even whole viral particles [25,26]. The transfer of specific functional proteins, mRNAs, microRNAs, and other biologically active molecules to uninfected cells can lead to significant changes in cellular processes. This can either promote further virus invasion or, conversely, impede infection by activating anti-viral defense mechanisms [27,28].

In this review, we analyze the role of extracellular vesicles in viral invasion and pathogenesis, and consider the data on EV-mediated regulation of antiviral immune response in a number of viral infections.

## 2. Biogenesis and Molecular Composition of Extracellular Vesicles

Initially, EVs were assigned the function of waste management: an effective and safe way for cells way to eliminate unwanted components, an alternative to their intracellular enzymatic degradation [29]. Subsequently, it was shown that EVs can perform more complex and specialized functions related to intercellular communication and signaling. The regulatory and communicative functions of EVs are realized through the intercellular transfer of numerous biologically active molecules: various types of nucleic acid, proteins, and lipids. The EV membrane acts as an effective protector from enzymatic degradation of these molecules in the extracellular medium. EV-transferred molecules impart new functional properties to cells by activating various signaling pathways and regulating gene expression.

Formation of different types of extracellular vesicles involves various cellular mechanisms. Biogenesis of exosomes (typical size 40–100 nm) is associated with the endosomal membrane compartment. Membranes of the late endosomes form secondary invaginations, and multivesicular bodies with multiple intraluminal vesicles are then generated [30]. Multivesicular bodies can subsequently merge with lysosomes, with degradation of their contents [31]. Alternatively, they can merge with the plasma membrane and secrete enclosed intraluminal vesicles as exosomes [32]. The most important molecular component of exosome formation is the endosomal sorting complex required for transport (ESCRT). It consists of four multi-protein complexes: ESCRT-0, ESCRT-I, ESCRT-II, and ESCRT-III [33]. ESCRT-0 is responsible for the clustering of proteins, which are ubiquitinylated during sorting and enter the intraluminal vesicles for further secretion in exosomes [34]. Protein TSG101 (tumor susceptibility gene 101) is a component of ESCRT-I that forms a complex with ubiquitinylated proteins and participates in the ESCRT-II activation, causing the formation of secondary invaginations in late endosomes [35]. ESCRT-II mediates the movement of ubiquitin-labeled proteins into intraluminal vesicles, acting in cooperation with ubiquitin-removing enzymes. The final stage of multivisicular body formation is the budding off of intraluminal vesicles, with the participation of ESCRT-III [36,37,38]. It is assumed that there is also an ESCRT-independent pathway of exosome formation, in which ceramides [39,40], tetraspanins, and heat shock proteins [41,42] play a crucial role.

Exosomes contain multiple specific proteins, nucleic acids, and lipids. According to data of high-throughput proteomic and transcriptomic analysis, over 2600 proteins, more than 900 mRNA, and more than 270 microRNAs are found in exosomes [43].

Unlike exosomes, another type of EVs, microvesicles, are directly budded from the plasma membrane [44]. On average, they are larger than exosomes (about 100–1000 nm). Components of future microvesicles are locally accumulated on the inner membrane side, then the membrane curves, forming a vesicle. This process is accompanied by specific translocase-mediated transfer of phosphatidylserine to the outer plasma membrane layer and contraction of the submembrane actin cytoskeleton [45,46]. Components of the ESCRT complex (TSG101 protein) are apparently involved in the formation of microvesicles as well [47].

Apoptotic bodies have a diameter of 500–5000 nm and contain highly compacted contents from the nucleus and cytoplasm. They bud off from the membrane during programmed cell death [48,49,50] and subsequently are captured by phagocytic cells (mainly macrophages) [51]. Briefly, the main molecules of extracellular vesicles are summarized in the Table 1.

**Figure 1:**
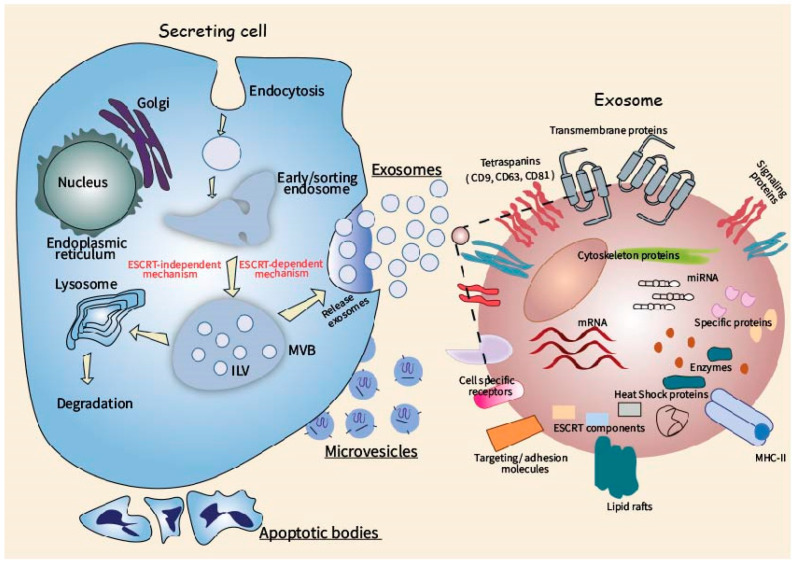
Cellular routes of extracellular vesicle (EV) biogenesis and main molecular components of the exosome.

The scheme of EV biogenesis and the main molecular components of exosomes, as the most fully characterized type of EV, are shown in Figure 1.

Interaction between EVs and target cells can occur by two different ways. First, EV membrane proteins can bind to receptor molecules on the surface of target cells, which are coupled to their signaling cascades. EV membrane proteins also can be cleaved by proteases and convert into soluble fragments, which act as ligands for corresponding cell receptors. Second, EV can fuse with the membrane of the target cells, with transfer of their cargo (protein, RNA) to the recipient cell [76].

## 3. Pro-Viral Role of Extracellular Vesicles in the Infectious Process

An objective analysis of EV effects on progression of viral infection meets certain methodological difficulties. Extracellular vesicles secreted by infected cells have very similar physical characteristics (size, buoyant density) to the majority of viruses. This makes the separation of viral particles and extracellular vesicles a difficult goal and complicates subsequent analytical studies [77]. Use of known protein markers to separate EV and viral particles is not always effective, because in reality not all vesicular structures can carry these markers on their surface [12]. On the other hand, various molecules of viral origin are selectively included into EVs, and this brings an additional complexity into EV separation and molecular analysis [78,79]. EVs of different origins were shown to carry a range of genetic material such as DNA fragments [80,81], mitochondrial DNA [82,83], and a great variety of RNAs including mRNAs, miRNAs, and small non-coding RNAs [84]. This ability makes EVs an important part of intercellular communication during virus infection. Transfer of nucleic acids derived by EVs from infected cells to the recipient could leads to proviral and antiviral effects as well [85].

Moreover, many viruses use pathways similar to EV pathways of biogenesis and secretion. This allows viruses to assemble with maximal efficiency in cells, with potent spreading to neighboring cells. In particular, many viruses use ESCRT and Rab GTPases for their secretion, namely, rhabdoviruses, filoviruses, arenaviruses, paramyxoviruses, herpesviruses, and hepatitis viruses A and C [86,87,88,89,90].

Extracellular vesicles are able to interact with the recipient cell via both receptor-dependent and receptor-independent pathways. Therefore, some viruses use EVs for their own invasion to expand the range of cells available for infection, which in general contributes to more efficient spread [91]. Overall, the role of EVs in viral pathogenesis is similar to that in tumor metastasis. Exosomes and microvesicles prepare the microenvironment for the further viral invasion.

Today the role of exosomes and other extracellular vesicles in the intercellular communication during cell infection is the most studied in the context of herpes simplex virus (HSV), Epstein-Bar virus (EBV), hepatitis viruses, and human immunodeficiency virus (HIV). Each of these viruses has unique characteristics and enhancing approaches of replication and spread. Virus adaptive and protecting systems are unique and effective as well. The use of extracellular vesicles containing specific cellular and viral components is one of the approaches implemented by viruses to achieve effective spread and infection of cells. A brief description of the main components of extracellular vesicles during different infections of viruses are presented in Table 2.

Below, we consider recent data on the molecular mechanisms of pro-viral exploitation of extracellular vesicles by a number of viruses investigated in this regard.

### 3.1. Herpes Simplex Virus (HSV-1)

HSV-1-infected cells secrete vesicular structures known as L-particles, which contain multiple cellular factors and viral envelope proteins (ICP0, ICP4) [112,113]. L-particles are comparable to exosomes in size; they are formed from the inner membranes of cells and are able to deliver their contents to neighboring non-infected cells [114]. The interaction of secreted L-particles with surrounding cells contributes to further viral spread and suppresses the immune response [115]. Though L-particles are non-infectious, they prepare cells for subsequent infection by activating the transcription of certain cellular proteins beneficial for subsequent HSV reproduction [116,117]. Moreover, the interaction of L-particles with mature dendritic cells reduces the expression of CD83 in them and ultimately contributes to immunosuppression [118].

In addition, HSV-1-infected cells secrete exosomes carrying viral glycoprotein B, which targets the MHCII synthesis pathway by antigen-presenting cells (APCs) [119]. In the cytoplasm of APCs, glycoprotein B binds to the HLA-DR protein receptor. This binding hinders HLA-DR receptor export to the cell surface. Thus, glycoprotein B reduces the number of HLA-DR receptors presenting viral determinants on the surface of APCs [120]. This also helps HSV-1 to avoid the immune response.

HSV-1 virions can be entirely included in microvesicles secreted by the infected cells, and later virions are transferred to neighbor cells. This allows the virus to expand the range of cells available for infection, as well as to avoid a targeted immune response [121]. The process of incorporation of HSV-1 virions into microvesicles is presumably associated with autophagy, since the autophagosome marker LC3-II has been found in microvesicles with viral particles [25].

### 3.2. Epstein-Barr Virus (EBV)

The gamma herpes virus EBV was the first oncogenic virus to be characterized [122], and its main oncogene is the membrane protein LMP1 [123,124]. This protein is selectively incorporated into exosomes secreted by EBV-infected cells [125]. To enter the exosomes, LMP1 interacts with tetraspanin CD63 [95,96] or is associated with lipid rafts [126]. Within exosomes, LMP1 is transferred to uninfected cells. LMP1-enriched exosomes also contain the viral glycoprotein gp350, which interacts with the CD21 receptor of B cells for exosome targeting [94]. Exosomal delivery of LMP1 to B cells causes their transformation [127]. LMP1 also induces massive apoptotic death of CD4^+^ T cells and natural killer cells [128].

Exosomes of EBV-infected cells selectively accumulate certain cellular proteins, such as galestine 9, which has a suppressive effect on Th1 T lymphocyte function [94,95]. In addition, exosomes deliver to uninfected cells a set of activated signaling proteins: epidermal growth factor receptor (EGFR); phosphatidylinositol-3-kinase (PI3K); fibroblast growth factor (FGF2); and HIF1-alpha [96,97,98,99,100,101]. These proteins trigger pathways responsible for recipient cell proliferation and tumor angiogenesis.

Exosomes of EBV-infected cells selectively include a variety of functional viral miRNAs and transfer them to recipient cells. More than 300 viral miRNAs (this is 20–25% of the entire fraction of cellular miRNAs) were detected in EBV-infected cells using NGS [129]. Numerous viral miRNAs, from all three known clusters (BHRF1, BART1, and BART2), were also identified during exosome profiling of EBV-infected cells [102,130]. Viral miRNAs are known to perform post-transcriptional multifunctional control, they can regulate infection pathogenesis (switching from lytic to persistent), induce changes in cellular metabolism beneficial to the virus, modulate the cellular life cycle, and evade host immune system action [131].

In particular, BHRF1 represses the IFN-inducible T-cell attracting chemokine CXCL11 [132], and miR-BART15 regulates NLRP3 inflammasomes and IL-1β production [133]. In addition, these miRNAs promote apoptosis in recipient cells by silencing the mRNA of apoptosis inhibitors such as BRUCE and TAX1BP1 [134,135]. Suppression of cytokine production, and the induction of apoptosis in immune recipient cells, contributes to the suppression of antiviral reactions against EBV. Thus, the exosomes of infected cells mediate immunosuppression and carry several signaling molecules (proteins and miRNAs) that create a favorable microenvironment for the progression of EBV-associated tumors, including Hodgkin’s lymphoma [136] and nasopharyngeal carcinoma [137].

Moreover, EVs secreted from EBV-infected cells contain coding and non-coding RNAs. Protein coding latent phase mRNAs (LMP1, LMP2, EBNA1, and EBNA2 gene transcripts) were detected in the exosomes from EBV-infected cells [138]. However, it is still unclear if these gene transcripts have biological activity after vesicular delivery into uninfected cells. EBV-encoded small RNAs (EBER1 and EBER2) are shown to be released from EBV-infected cells. They are suspected of supporting the survival and carcinogenesis of infected cells by avoiding cell apoptosis via signaling from TLR3 [139].

### 3.3. Human Immunodeficiency Virus (HIV)

For HIV, the “Trojan horse hypothesis” was formulated, postulating a hijacking, by HIV, of the pathways of exosome biogenesis and secretion to assemble and to bind the target cell without Env viral protein participation [140]. High similarity in lipid composition (high cholesterol and glycosphingolipid content) and protein composition (tetraspanins, GPI proteins, some cytoplasmic proteins) of exosomes and retroviruses was observed [141]. HIV also uses ESCRT complex components for self-assembly and release. Inhibition of exosome secretion in infected cells leads to a partial decrease in the virion release of HIV progeny [142,143]. However, the prevailing route of HIV progeny release is budding from the plasma membrane (similar to microvesicles) in regions with a high Gag protein level [144,145,146]. Therefore, the detailed molecular mechanism of the contribution of the EV biogenesis pathway to HIV replication remains unclear.

The main factor of CD4^+^ T cell apoptosis during HIV infection is the viral protein Nef. There is clear evidence that it is exosomes that mediate intercellular Nef transport to uninfected T cells, with their subsequent apoptotic death [105,147]. The Nef uptake by exosomes is based on their interaction with mortalin (Hsp70 family member) [148]. Nef-containing vesicles can also be captured by B cells, contributing to decreased IgG2 and IgA production and suppression of humoral immune response [149]. In addition, Nef enhances the production of multivesicular bodies and EV secretion [149,150,151], thereby enhancing the exosomal spread of this virulence factor. EVs can also transfer the HIV coreceptors CCR5 and CXCR4 to uninfected cells, which do not yet have those proteins. This process significantly facilitates further spread of infection [106,107]. Other viral components transported into EVs from HIV-infected cells are Gag protein (its impact on infectious spread is still unclear) [103] and gp120 envelope protein, which seemed to increase significantly the viral infectivity in human lymphoid tissues [104].

Moreover, exosomes secreted from HIV-infected cells are capable of carrying RNA, in particular HIV transactivation response element (TAR), which also increases the susceptibility of cells to viral infection [108]. The hairpin (stem-loop structure) at the 5’-end of TAR RNA binds to the Tat protein (trans-activator of transcription) in infected cells, enhancing viral RNA transcription [152].

### 3.4. Hepatitis C Virus (HCV) and Hepatitis A (HAV)

Hepatitis C virions have a size about 50 nm and are assembled in the cytoplasm of infected cells with the participation of components of exosome biogenesis [153]. EVs of infected cells contain viral E1 and E2 proteins [109] and viral RNA. The exosome marker CD81 is associated with viral proteins, which indicates a possible CD81 role in the packaging of viral proteins into exosomes [154]. Vesicles carrying the E2-CD81 complex increase the infectivity of the hepatitis C virus (HCV). EVs from infected cells can also contain whole virions [155]. Delivery within extracellular vesicles protects the virus from attack by neutralizing antibodies, thus contributing to the evasion of the immune system [156]. It also allows HCV to enter an increasing variety of potential host cells (other than hepatocytes) upon receptor-independent fusion of EVs with the cell membrane. Exosomes from infected cells are also capable of transferring viral RNA in complex with miR-122, Ago2, and HSP90, ensuring more efficient viral replication [110].

In contrast to HCV, the hepatitis A virus (HAV) is non-enveloped, but it is able to be incorporated into EVs for secretion and spread as well due to small virion size. Virion packaging into the vesicles is a result of interaction between the structural viral protein pX and the Alix protein [111]. The use of the cellular ESCRT complex allows the viral particles to be released into the extracellular space without rupture of the cell. Thus, non-enveloped HAVs acquire an envelope made from the host cell membrane, with no transmembrane viral proteins [25,26]. Usually, during HAV infection, virions are detected in feces. However, viral particles in EVs can circulate in the blood and will not be attacked by neutralizing antibodies. In addition, packaging of viral particles inside EVs increases the likelihood of infecting cells other than hepatocytes, in particular the cells of spleen or lymph nodes, which filter blood and lymph.

### 3.5. Coronaviruses

Viruses of the family Coronaviridae are enveloped, 80–120 nm in size, with a viral genome consisting of large single-stranded positive RNA [157]. Some coronaviruses are known to be etiological agents of dangerous pandemic respiratory illnesses such as SARS [158], MERS [159], and COVID-19 [160]. MERS-CoV infection is associated with interaction between the viral S-protein and cell dipeptidyl peptidase-4 (DPP4) [161,162]. Other cellular cofactors for viral infection are tetraspanins. In particular, CD9 tetraspanin contributes to a more effective infectious process and leads to more severe infection in mice [163]. CD9 is a major protein of exosomes. Therefore, it can be assumed that exosome biogenesis pathways are involved in MERS-CoV infection.

Other CoV proteins are also involved in membrane modifications and can be found in EVs from infected cells. The S2-glycoprotein subunit possesses various membranotropic segments that induce membrane perturbation and could allow membrane negative curvature [164]. In addition, it was reported that M and E glycoproteins of CoV can mediate the formation and release of 100 nm “vesicles”, which are morphologically very similar to viral particles. For other viruses, the production of such vesicles greatly assists infection spread, masking real virions from immune response [165].

The SARS-CoV and SARS-CoV2 viruses use angiotensin-converting enzyme 2 (ACE2) as a receptor [166,167]. ACE2 is abundant on the surfaces of lung alveolar type II (AT2) cells, and it is also expressed in the heart, blood vessels, liver, digestive organs, and kidneys [168]. Exosomes are able to carry ACE2 [169] and deliver it to uninfected recipient cells, presumably making them susceptible to the infection. Thus, exosomes can contribute to coronavirus pathogenesis. However, clarification of exosomes’ roles in coronavirus infections requires further research.

Recent, electron microscopy-based research has demonstrated that virions of SARS-CoV and SARS-CoV2 are associated with double membrane-enveloped vesicles in infected cells [170,171]. This may be indicative of the exploitation of cellular systems for EV biogenesis by coronaviruses for their spread and evasion of the immune system, like that seen in a number of other viruses.

## 4. EVs in Antiviral Defense

Extracellular vesicles not only participate in viral pathogenesis, but also can help the host’s organism to resist the infection. There is a lot of evidence that secretion of exosomes and microvesicles activates antiviral immune responses due to various molecular signals contained in the vesicles [172,173,174]. Exosomal proteins are one of these signals. Epithelial cells infected with cytomegalovirus are known to secrete EVs containing viral antigens. These antigens are delivered by EVs to antigen-presenting cells, with subsequent immune response activation [175]. Similarly, with influenza A virus, exosomes transfer hemagglutinin epitopes in complex with MHCII molecules, increasing the efficiency of antigenic determinant presentation to immune cells [176]. Macrophages infected with influenza virus secrete exosomes containing a wide range of specific proteins: proteins binding fatty acids, proteins of copper metabolism, cytokines (IFN-α1, IFN-α14, IFN-β, IL-6, IL-18, TNF), and proteins involved in cellular autophagy [177]. Additionally, EVs derived from virus-infected dendrite cells are implicated in the stimulation of proliferation and differentiation of CD8+ T cells into effector cytotoxic T lymphocytes (CTLs). This occurs in induction of immune responses to tumors and in protecting neighbor cells from tumor growth [178].

RNAs contained in exosomes during viral infection can also be a signal for immune activation. The exosomes from infected cells include both viral mRNAs and microRNAs. Upon transfer to other cells, viral RNA is recognized by pattern recognition receptors that signal the organism about viral attack and trigger innate immunity reactions. Viral RNAs have been found within exosomes derived from HCV, HIV, EBV, and cytomegalovirus (CMV)-infected cells. For example, exosomes from HCV-infected cells transfer viral RNA to plasmacytoid dendritic cells, stimulating the production of IFN-α [179]. EVs secreted from HIV-infected cells carry viral RNAs, which stimulate Toll like receptor-8 (TLR8) signaling to promote TNFα release, contributing to chronic immune activation [180]. Another well-known example is EBV that exploits different types of RNAs engaged in vesicles to block the antiviral response. Viral proteins encoded with mRNAs gene transcripts that play key roles in the latent infectious phase were found in the exosomes from EBV-infected cells [138]. Several small non-coding RNAs (EBER1 and EBER2) have been found in EVs secreted from EBV-infected cells, they are suspected of supporting the survival of infected cells by avoiding cell apoptosis [139]. In addition miRNAs (miR-BART15-3p) presented in exosomal cargo from EBV-infected cells were shown to induces apoptosis in immune cells [134]. In addition, profiling of EBV-induced lymphoblastoid cell lines revealed that long non-coding RNAs H19 and H19 antisense exist in secreted exosomes, and they are suggested to play a role in the regulation of the microenvironment of cells [181]. In the case of cytomegalovirus (CMV)-infected cells, the herpes simplex virus 1 (HSV-1) secreted exosomes containing viral mRNAs and microRNAs, whose major function may be in silencing viral genes in latently infected neurons. Moreover, this pull of RNAs is presented in exosomes rather than in virions. It has been suggested that HSV-1 controls the spread of infection between cells using exosomal RNAs for curtailing the infection under certain conditions [182].

HIV-infected macrophages secrete extracellular vesicles containing viral miRNA, which stimulates TLR8 and NF-κB signaling pathways when passed to other macrophages. As a result, the production of proinflammatory cytokines (for example, TNF-α) is enhanced, and the immune response against HIV is activated [180]. Cells infected with HSV-1 secrete exosomes containing the viral miRNAs miR-H28 and miR-H29. Inhibition of viral replication and spread has been observed after exosomal transfer of these microRNAs to neighboring uninfected cells [183].

Numerous microRNAs of cellular origin are also contained in exosomes and transferred by them from infected to intact cells, triggering an antiviral response. For example, exosomes derived from the bronchoalveolar lavage of patients infected with the influenza virus accumulate miR-483-3p, mir-374-5p, and miR-446i-5p. These microRNAs promote the expression of IFN-β, proinflammatory cytokines, and interferon-stimulated genes, including IL-6, TNF-α, C-C motif chemokine ligand 2 (CCL2), and SP100 [184]. Another example is the high resistance to viral infection in trophoblast cells, which protects the fetus from infection. These cells secrete exosomes containing a specific set of cellular microRNAs (chromosome 19 miRNA cluster), and transfer of these microRNAs to recipient cells makes them less sensitive to a wide range of viruses [185].

Besides various molecular signals, exosomes from infected cells also contain effector molecules, directly participating in the antiviral host response. It is known that CD4^+^ T-cells secrete specific extracellular vesicles containing CD4 (CD4 is an HIV receptor). These EVs are able to bind HIV virions, ultimately decreasing the infection. HIV counteracts this defense mechanism by loading its Nef protein into the EVs secreted by infected cells. Nef, being delivered to T cells, prevents the incorporation of CD4 into EVs and suppresses the antiviral response described above [186].

Exosomes isolated from HIV-infected cells were also shown to carry cytidine deaminase, which inhibits viral replication in cells [187]. Cytidine deaminase APOBEC3G is incorporated into virions and causes deamination of cytidine in the synthesized single-stranded DNA during reverse transcription of viral RNA [188]. Structural remodeling of the synthesized DNA further leads to G-A point mutations. However, the APOBEC3G effect can be blocked by the viral protein Vif, which binds APOBEC3G and promotes its ubiquitinylation, with subsequent degradation in proteosomes [189].

Exosomes derived from respiratory tract epithelial cells during influenza A infection carry mucins (MUC1, MUC4, MUC16) [190], which are bound to sialic acids and are capable of virus neutralization. With Dengue virus, infected cells secrete exosomes with the effector antiviral protein IFITM3. IFITM3-containing exosomes are transferred to uninfected cells and equip them before they face the viral invasion [191].

One of the promising approaches to COVID-19 therapy is the use of multipotent mesenchymal stromal cells (MMSCs), which can reduce the cytokine storm in patients with severe disease [192]. After systemic injection, some of the MMSCs accumulate in the lungs, helping to restore their functionality. MMSCs are resistant to viral infection because they actively express IFN-stimulated genes (ISGs) and do not carry ACE2 (SARS-CoV-2 receptor) on their surfaces [193]. MMSCs affect innate and adaptive immune cells (including T-cells, B-cells, dendritic cells, macrophages, and natural killer cells) by secreting a wide pool of mediators of various types [194,195]. In addition, MMSCs secrete extracellular vesicles containing these mediators [196]. This gave rise to the idea of therapeutic usage of MMSC-derived EVs, instead of MMSCs themselves [197,198,199]. This is much safer and more practical, compared to MMSC-cell therapy; development of inhalational preparations is possible in this case.

A widespread complication of SARS-CoV-2 is myocardial injury, including ischemia and heart attack [200,201]. Treatment with MMSC exosomes has been experimentally proven to reduce infarction damage, to reduce oxidative stress, and to increase levels of NADH and ATP. Apparently, this is due to activation of the PI3K/Akt signaling pathway responsible for cell viability after myocardial injury [202,203]. Exosomes carrying the S-protein were also investigated as a candidate SARS-CoV vaccine in vivo. After injection, a significant titer of neutralizing antibodies against the S-protein in blood was detected in mice [204].

Thus, detailed studies of EV biology are necessary for understanding the mechanisms of exploitation of cellular systems of biogenesis and secretion of EVs by viruses and the deregulation of cellular processes under their influence. Multiple similarities between EVs and viruses in their organization, assembly, and molecular composition suggest approaches for development of new generation vaccines and biomimetic designs for targeted drug delivery [205]. In particular, COVID-19 treatment with MMSC-derived exosomes is a promising new approach. It requires, however, much more research to standardize isolation conditions, composition, storage, and dose selection depending on illness severity.

## 5. Conclusions

Analysis of the roles of extracellular vesicles during viral infection is extremely important for understanding the mechanisms of pathogenesis and possible ways to resist viral infection. Viruses can use the EV biogenesis system to activate their own secretion or to package viral material into vesicles. EV-mediated transfer of viral proteins and nucleic acids (including whole genomes and completely assembled viral particles) enhances viral spread and provides viruses with evasion of the immune response [205]. In addition, extracellular vesicles are capable of delivering surface receptors to uninfected cells for subsequent binding with viral particles, which leads to an increasing number of cells sensitive to viral infection. On the other hand, transfer and presentation of individual viral components to immune cells by EVs can trigger antiviral reactions. Exosomes could be good therapeutic agents, because they are non-immunogenic and can pass the cellular barriers, and the contents can be manipulated. EVs are capable of transporting both hydrophobic (inside) and hydrophilic (in the compound of the lipid membrane) molecules [206]. However, EVs are not optimized for the encapsulation of hydrophilic molecules. The loading capacity of the exosomes is rather low due to the presence of proteins and nucleic acids within. To improve cell targeting, exosomes should be generated by cells expressing ligands with a high binding affinity to the target cells [207,208]. However, there are some reported positive results in implementing exosomal delivery for therapeutics against neurotropic viruses [209]. On that basis, EVs are considered a promising component for use in new generation vaccine designs [210].

## Figures and Tables

**Table 1:** Molecular components of extracellular vesicles.

	Proteins	Lipids	Nucleic Acids
Exosomes	Main ESCRT proteins TSG101 [35] Alix [52] Rab proteins [53] Annexin A2 [54] Target cell recognition and fusion proteins tetraspanins CD9, CD63, CD81 [55] integrins and connexins [56] Cytoskeletal proteins actin, tubulin, cofilin [46] Heat shock proteins HSP60, HSP70, HSP90 [57] Proteins regulating immune responses Fas ligand (FasL), tumor necrosis factor alpha (TNF-α), transforming growth factor beta (TGF-β) [58] Enzymes Phosphoglycerate kinase 1 (PGK1), glyceraldehyde3-phosphate dehydrogenase (GAPDH) [55]	Higher content then in cells Cholesterol Ceramides Sphingomyelin Glycosphingolipids Phosphatidylserine (PS) [59] Similar content Phosphatidylethanolamine (PE) Lower content then in cells Phosphatidylcholine (PC) Phosphatidylinositol (PI) Diacylglycerol [60,61] +Biologically active lipids Prostaglandins, lysophospholipids [62]	mRNAs non-coding RNAs microRNAs [63,64,65,66] Genomic and mitochondrial DNA [67] tRNA fragments [68]
Microvesicles	Membrane proteins Integrins and flotilin-1, matrix metalloproteinase MT1-MMP, glycoprotein receptors (GP1b and GPIIb/GPIIa), adhesion protein P-selectin [69]	Cholesterol, sphingomyelin, ceramide [70], Phosphatidylserine Polyunsaturated lipids [71]	miRNAs, mRNAs non-coding RNAs [72], genomic and mitochondrial DNA [73]
Apoptotic bodies	Marker proteins Annexin V [74] Thrombospondin C3b Cytoplasmic proteins	Limited by the plasma membrane	rRNAs [24] Genomic and mitochondrial DNA [75]

**Table 2:** The main components of extracellular vesicles, secreted from cells infected by different viruses.

Herpes simplex virus (HSV-1)	Viral tegument proteins and other glycoproteins [92] Viral glycoprotein B [93]
Epstein-Barr virus (EBV)	LMP1 [94,95] Galestine 9 [96,97] Epidermal growth factor receptor (EGFR) [98] Phosphatidylinositol-3-kinase (PI3K) [99] Fibroblast growth factor (FGF2) [100] HIF1-alpha [101] Viral RNAs (BHRF1, BART1, and BART2) [102]
Human Immunodeficiency Virus (HIV)	Gag proteins [103,104] Nef protein [105] HIV coreceptors CCR5 and CXCR4 [106,107] Transactivation response element (TAR) [108]
Hepatitis C virus (HCV)	Viral E1 and E2 proteins [109] Viral RNA [110]
Hepatitis A (HAV)	Viral particles [111]
